# Supplementary material for: Content-rich biological network constructed by mining PubMed abstracts
Source: BMC Bioinformatics. 2004 Oct 8;5:147. doi: 10.1186/1471-2105-5-147 (PMC528731; doi:10.1186/1471-2105-5-147)
Supplement: Additional File 2 — The original results of the above study (non-essential files are deleted to keep the file size under the limit set by BMC bioinformatics). [file 1471-2105-5-147-S2.bz2 › chilibotAdditionalFile2/dip30/370ID9045692E6045/html/FGF7_FGFR3.html]

 


 **FGF7** and **FGFR3** 
  
Found 22 abstracts in PubMed, retrieved 22.  
 

 What does Google say? 
 PDF only 
| .edu only 

---

**Interactive relationship** (e.g. stimulation, inhibition, etc)

**Neutral relationship**- Furthermore, by constructing a chimeric receptor that contains the homologous exon from FGFR2, we demonstrate that this single domain from FGFR2 is sufficient to confer upon  **FGFR3**  the ability to bind KGF  [ **FGF7** ]  FGF 7  [ **FGF7** ] .  Ref: 7512569 J Biol Chem, 1994
- Prostatic epithelial cells express primarily the FGFR 3  [ **FGFR3** ]  IIIc isoform, which preferentially binds FGF1 over FGF2, with smaller amounts of the FGFR 2 IIIb  **FGF7**  binding isoform.  Ref: 8976296 J Urol, 1997

**Non-interactive relationship** (e.g. studied together, co-existance, homology, etc.)

- Reverse transcriptase polymerase chain reaction analysis of human keratinocyte mRNA for fibroblast growth factor receptors FGFRs revealed expression of only FGFR 2 IIIb, the KGF  [ **FGF7** ]  specific receptor, which also binds bFGF with low affinity, and FGFR 3  [ **FGFR3** ]  IIIb, which does not bind bFGF or KGF  [ **FGF7** ] .  Ref: 9218449 J Biol Chem, 1997
- METHODS The expression patterns of FGFR2 bek and keratinocyte growth factor  [ **FGF7** ]  receptor KGFR variants and  **FGFR3**  were analyzed by in situ hybridization during embryonic and postnatal lens development.  Ref: 9286257 Invest Ophthalmol Vis Sci, 1997
- Keratinocyte growth factor  [ **FGF7** ]  KGF  [ **FGF7** ] , a ligand of another member of the FGF receptor family, enhanced expression of  **FGFR3**  IIIb, but acidic FGF, the ligand for  **FGFR3**  IIIb itself, had no effect.  Ref: 9142922 Am J Physiol, 1997
